# Fast Sensing of Hydrogen Cyanide (HCN) Vapors Using a Hand-Held Ion Mobility Spectrometer with Nonradioactive Ionization Source

**DOI:** 10.3390/s21155045

**Published:** 2021-07-26

**Authors:** Victor Bocos-Bintintan, Ileana Andreea Ratiu

**Affiliations:** 1 Faculty of Environmental Science and Engineering, Babes-Bolyai University, RO-400294 Cluj-Napoca, Romania; 2 Transcend SRL, RO-400568 Cluj-Napoca, Romania; 3 “Raluca Ripan” Institute for Research in Chemistry, Babes-Bolyai University, RO-400294 Cluj-Napoca, Romania

**Keywords:** hydrogen cyanide (HCN), ion mobility spectrometry (IMS), trace detection, toxic industrial compounds (TICs), chemical warfare agents (CWAs)

## Abstract

Sensitive real-time detection of vapors produced by toxic industrial chemicals (TICs) always represents a stringent priority. Hydrogen cyanide (HCN) is definitely a TIC, being widely used in various industries and as an insecticide; it is a reactive, very flammable, and highly toxic compound that affects the central nervous system, cardiovascular system, eyes, nose, throat, and also has systemic effects. Moreover, HCN is considered a blood chemical warfare agent. This study was focused toward quick detection and quantification of HCN in air using time-of-flight ion mobility spectrometry (ToF IMS). Results obtained clearly indicate that IMS can rapidly detect HCN at sub-ppm_v_ levels in air. Ion mobility spectrometric response was obtained in the negative ion mode and presented one single distinct product ion, at reduced ion mobility K_0_ of 2.38 cm^2^ V^−1^ s^−1^. Our study demonstrated that by using a miniaturized commercial IMS system with nonradioactive ionization source model LCD-3.2E (Smiths Detection Ltd., London, UK), one can easily measure HCN at concentrations of 0.1 ppm_v_ (0.11 mg m^−3^) in negative ion mode, which is far below the OSHA PEL-TWA value of 10 ppm_v_. Measurement range was from 0.1 to 10 ppm_v_ and the estimated limit of detection LoD was *ca*. 20 ppb_v_ (0.02 mg m^−3^).

## 1. Introduction

After its inception in the 1970s, ion mobility spectrometry (IMS) became a mature analytical technique, widely used nowadays for quick trace detection and quantification of vapors after their ionization at atmospheric pressure [1]. The crucial advantages of IMS lie in its very rapid response, the outstanding sensitivity, and the simple, very robust, and compact instruments. IMS is being utilized today for a broad range of applications—detecting traces of energetic materials, illegal drugs [2,3], precursors of illegal drugs [4,5], chemical warfare agents (CWAs), and toxic industrial chemicals (TICs) [6,7,8], plus a continuously expanding number of biomedical and industrial applications [9,10]. Moreover, the IMS technique was found to be useful in other fields too, such as medicine, forensic analyses, industrial hygiene, biology, space research, and investigation of the environment [1]. In addition, a very challenging application is related to sensing and discriminating various strains of microorganisms [11,12].

IMS is based upon the separation of ions—generated by the ionization of the neutral chemical species in gaseous phase at atmospheric pressure—due to their different masses and shapes—in a relatively weak (<500 V/cm) electric field. In ToF (time-of-flight) IMS, separation of ions occurs because they have different mobilities when travelling through a neutral drift (buffer) gas, which is usually either nitrogen or air, under the propelling force exerted against them by the longitudinal DC electric field. Therefore, one may claim that in IMS the ions produced are subjected to atmospheric pressure time-of-flight measurements. Using an ionization source in air, in ToF IMS, a radioactive ionization source initiates a very complex and fast ion–molecule reaction chemistry that leads initially to the formation of ion clusters called “reactant ions” of type (H_2_O)_x_H^+^ (predominant), (H_2_O)_y_NH_4_^+^, and (H_2_O)_z_NO^+^ in the positive ion mode, or the (H_2_O)_n_O_2_^−^ species in the negative mode. Water vapor plays a crucial role in the atmospheric pressure ion–molecule chemistry, so it is necessary to continuously control and maintain its concentration inside the IMS measurement cell at a constant low level [7,8].

Hydrogen cyanide HCN is considered both a toxic industrial chemical (TIC) and a chemical warfare agent (CWA). HCN is used in large amounts as a commercial industrial compound, for instance, for manufacturing synthetic fibers (nylon, methyl methacrylate, acrylonitrile) and many other synthetic materials, such as insulating foams, adhesives, cleaning products, drugs, food additives, paints and varnishes, lubricants, plastics, dyes, and pesticides. The major uses of hydrogen cyanide are therefore as a reagent in the production of a large number of chemicals and as an insecticide. HCN is also utilized in petroleum refining, electroplating, metallurgy, and photographic development [13]. Hydrogen cyanide is also generated during fires, when various nitrogen-containing polymeric materials are burnt [14].

As a CWA, HCN (military designation AC) is a nonpersistent chemical agent, due to its high volatility. Being a systemic chemical asphyxiant, HCN is a “blood agent” and has been used so far as a war gas, in the genocidal Nazi concentration camps, in terrorist attacks, and in judicial executions in some states of the USA. Poisoning potential of HCN is reflected by the low lethal dose LD_50_, which is 50…60 mg HCN for oral uptake and 1 mg intravenous, for a 75 kg person [15].

HCN has a linear polar molecule and is a colorless to pale-blue liquid that vaporizes very quickly, producing therefore potential lethal concentrations in close rooms. Hydrogen cyanide vapors are slightly lighter than air (relative density of vapors is 0.94, air having 1) and have a faint odor of (bitter) almonds. In addition, vapors of HCN are flammable and potentially explosive above 5.6% (*v*/*v*). Its solubility in water is high, so it can be used as a 96% solution in water. Volatility of HCN is very high, which is reflected by its vapor pressure of 742 Torr (25 °C). HCN is very flammable, has a high acute toxicity, and in some conditions, its vapors may decompose explosively [13]. Conversion is 1 ppm_v_ = 1.12 mg m^−3^ (20 °C).

Exposure to HCN concerns both workers and general population. Workers who use HCN may be exposed by breathing it as mists/vapors or by direct skin contact; the greatest exposure to HCN is among fumigant workers, organic chemical synthesizers, electroplaters, gold and silver extractors, steel workers, and workers in the plastics industry, in particular for manufacturing the acrylonitrile–styrene copolymer [16]. The general population may be exposed to HCN mainly by smoking cigarettes and by ingestion of some foods containing hydrogen cyanide as flavoring agent [13].

Toxicity of HCN is due to the disruption realized to respiration processes at a cellular level. Poisoning with HCN is fatal mainly following vapor inhalation, but also through oral or dermal exposures, because of the severe depression of the central nervous system. In addition, HCN is an environmental hazard, being very toxic for aquatic life, with long-lasting effects [13].

For HCN, the OSHA permissible exposure limit for 8-h time weighted average (PEL-TWA) is set at 10 ppm_v_ (*ca*. 11 mg HCN m^−3^ of air), while the threshold limit value (TLV) is 4.7 ppm_v_ (5 mg m^−3^). NIOSH recommendations are the short-term exposure limit (REL-STEL) is 4.7 ppm_v_ (5 mg m^−3^)—skin; PEL-TWA is 10 ppm_v_ (11 mg m^−3^)—skin, and immediately dangerous to life or health (IDLH) level is 50 ppm_v_ (56 mg m^−3^). Acute exposure guideline levels (AEGLs) from EPA range from 1 ppm_v_ for 8 h (causing discomfort) to 27 ppm_v_ for 10 min (life-threatening/death) [17]. An exposure for several minutes to 300 ppm_v_ HCN may result in death, while an exposure to 150 ppm_v_ for 0.5 … 1 h may endanger life. 

It is axiomatic that, given the high acute toxicity of hydrogen cyanide, maximum care has to be taken in order to reduce exposure to this chemical compound—especially with the goal of protecting humans. Consequently, fast, compact, lightweight, and sensitive analytical systems able to detect HCN at trace levels (in the ppm_v_ and sub-ppm_v_ range) are stringently required.

Detection and quantification of HCN present in air samples can be currently performed using a diversity of analytical methods, such as VIS spectrophotometry, gas chromatography coupled with mass spectrometry (GC/MS), liquid chromatography in tandem with mass spectrometry (LC/MS), mass spectrometry, and electrochemical methods (e.g., potentiometry using cyanide-specific electrodes, ion chromatography, or direct current amperometry), with limits of detection in the range of μg or <1 ppm_v_ [13].

We have proven through this study that low-resolution, hand-held ToF IMS instrumentation with nonradioactive (corona discharge) ionization source is perfectly fit for quickly sensing and quantifying vapors of HCN at ultra-trace levels, while offering a series of additional strategical advantages over the vast majority of available analytical technologies: the fast, real-time response (occurring in just several seconds), high sensitivity (with a minimum measured level of 100 ppb_v_ HCN), and good selectivity due to the monitoring of negative ions. Our results are also discussed and compared with other researchers’ findings. 

The scientific novelty of our work relies on (A) performing a thorough investigation of the analytical figures of merit concerning IMS detection of HCN at very low concentration levels, and (B) producing of standard atmospheres starting from a certified reference material with 10 ppm_v_ HCN—unlike other approaches, where chemical generation of HCN has been used.

## 2. Materials and Methods

### 2.1. The IMS Instrument

A hand-held portable time-of-flight (ToF) IMS instrument (size: 18.0 cm × 11.5 cm × 4.5 cm; weight: *ca*. 0.6 kg, with the set of four alkaline AA batteries and the molecular sieve air filter included), providing a real-time response (in several seconds), model LCD-3.2E, manufactured by the company Smiths Detection Ltd., Watford, U.K., was used for detection and quantification of HCN vapors. The IMS cell has, in principle, a classic design with stacked rings, where the conducting rings are discrete and positioned alternately onto the insulating body of the IMS measuring cell. The drift length of the IMS cell is *ca*. 30 mm, while the electric field intensity is about 270 V cm^−1^. Operating temperature of the IMS cell is around 25 °C, while the pressure inside the cell is atmospheric pressure (*ca*. 1000 mbar). The ionization source is a nonradioactive one, using a corona discharge in point-to-plane configuration. As drift gas, purified dry air is used, which is recirculated in a closed-loop pneumatic circuit that contains a filter with 10A molecular sieve; filter material incorporyesates and also delivers controlled levels of ammonia NH_3_, which functions as a dopant that greatly enhances selectivity in positive ion mode. This lightweight chemical detector, model LCD-3.2E, is, most probably, the smallest commercial ToF IMS instrument that exists on the market and is perfectly able to detect both TICs and CWAs within seconds. The LCD-3.2E instrument uses two parallel drift cells; it has, therefore, a “twin IMS cell” configuration, which offers the significant advantage of generating simultaneously both positive and negative ion mobility spectra.

**Figure 1:**
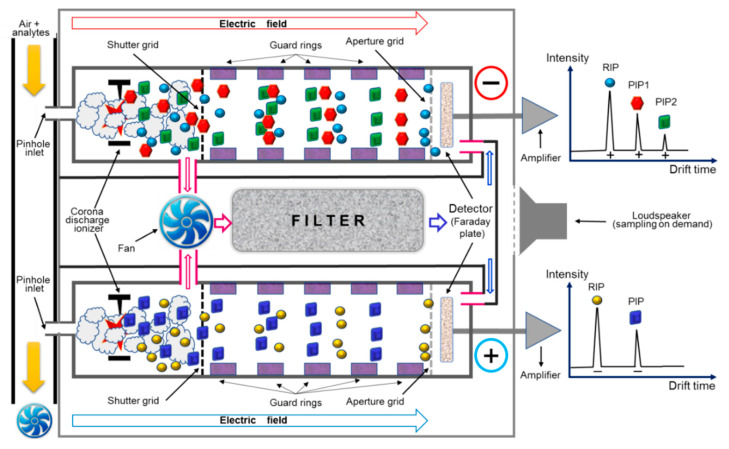
Schematic of the portable ToF IMS instrument model LCD-3.2E used for experiments.

Sample air is drawn from the inlet into the sampling line using a fan having a flow rate of about 1.0 L min^−1^; after that, a small volume of air sample is introduced into the two ionization regions through two pinhole inlets using a reduced internal pressure produced by the vibrational movement of a diaphragm (which is a loudspeaker-type pressure pulser). Finally, the residual air sample is exhausted outside. Ionization of the sample molecules is realized by the corona discharges in the two ionization regions, and this way, both positive and negative product ions are simultaneously produced by reaction with the afferent reactant ions. Ionization regions are further connected with their corresponding ion drift (separation) regions, which are kept at ambient temperature; purified drift gas (air containing ammonia dopant, emitted from the sieve pack) is continuously circulated in the opposing flow mode with moving ions. The positive and negative ions are periodically injected into their drift regions by a gating (shutter) grid. The inner air that circulates inside the drift regions and ionization regions is continuously passed through the purifying sieve pack by using a fan as a pump. Periodical exchange of the sieve pack filter is, of course, needed when the water content increases too much and, thus, the position (drift time) of the reactant ions’ peak shifts towards longer drift times due to the increase in water clustering [6,7]. Both the positive and negative scans were performed, every several seconds, in order to record the ion mobility spectra of 20 ms each. For our experiments, the power supply interface (mains, AC 220 V/50 Hz) was also housing the computer interface. The instrument was operated via a PC computer, using the proprietary IMS control software TrimScan2, ver. 0.4.0 (Smiths Detection Ltd.). This way, data (as negative ion mobility spectra) were saved on the hard disc as IMS spectra, which were subsequently analyzed using the aforementioned proprietary software, TrimScan2. A schematic of the lightweight chemical detector ToF IMS model LCD-3.2E, including the typical resulting responses (ion mobility spectra), is presented in Figure 1.

The LCD-3.2E instrument uses a corona discharge, nonradioactive ionization source. Corona discharge is currently one of the very few viable alternatives to the classical, almost ubiquitous radioactive sources (based on beta radiation isotopes, such as 63-Ni or 3-H) that equip currently the ToF IMS devices. Having ionization chemistry pretty similar to standard radioactive sources, a corona discharge ionization source yields, in most instances, a higher response, meaning a larger ion current, usually with an order of magnitude. Since the electrons’ density is higher, electron capture by electronegative species (such as cyanide) has a good efficacy; hence, the sensitivity towards electronegative compounds is expected to be increased compared to a classical radioactive source. 

The key feature of IMS behind the amazing capabilities of ToF IMS is fast separation of gas-phase ions under the influence of a low intensity electric field; ions are being generated in a soft manner—first, the reactant ions are formed, then charge is transferred to the molecules of analyte and thus the so-called “product ions” that include the whole molecule of target analyte eventually result. Both positive and negative ions are generated in an IMS cell, hence either a positive or a negative ion mobility spectrum will be obtained. A single ion mobility spectrum, typically recorded in only 20 ms, looks very similar to a chromatogram (Figure 1); therefore, drift time has practically the same role that retention time has in chromatography, being a qualitative parameter highly relevant in identifying a certain compound.

All measurements were taken after replacing the standard rain cap with the survey nozzle, in order to realize an optimal air sample transfer inside the IMS instrument.

In ToF IMS, the constant drift speed of any ion travelling through the drift region is v_d_ = K·E = l_d_/t_d_ (where K is ion mobility, l_d_ is drift length, and t_d_ is drift time), so that for low field conditions valid in any ToF IMS instrument, K = v_d_/E = l_d_/(E⋅t_d_). By normalizing ion mobility for temperature and for pressure, the “reduced” ion mobility K_0_ = K·(T_ambient_/T_cell_)·(P_cell_/P_atmospheric_) is obtained, which is regarded as an useful qualitative parameter that characterizes a substance [1]. Ion mobility depends on ion mass and its charge, but also on ion shape and size.

### 2.2. Sampling and Work-Flow Procedure

**Figure 2:**
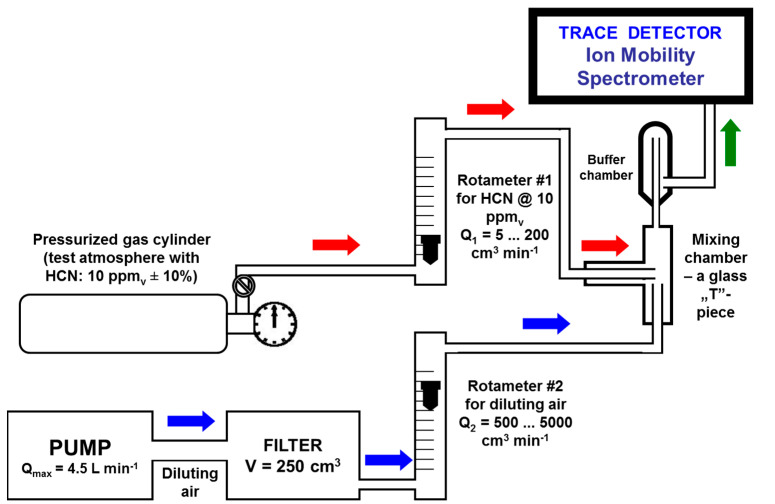
Schematic of dynamic system used for generation of test atmospheres with HCN.

A gas cylinder containing a standard atmosphere of 10 ppm_v_ HCN (±10%) in nitrogen, from RAE Systems Inc., Sunnyvale, CA, USA, was used. Test standard atmospheres at trace levels (ppm_v_ and sub-ppm_v_) with known concentrations of hydrogen cyanide were prepared by using the dynamic method of single-stage gas flow mixing. In other words, in order to obtain the investigated test atmospheres, a small flow rate (from 5 to 200 cm^3^ min^−1^) of gas containing 10 ppm_v_ HCN vapors was mixed with a larger flow (from 500 to 5000 cm^3^ min^−1^) of clean diluting air. The two rotameters were previously calibrated. A schematic diagram of this homemade dynamic test atmosphere generator (TAG) used for generation of standard atmospheres with very low concentrations of HCN vapors is presented in Figure 2. Diluting air flow was purified using a 10A molecular sieve filter. The air pump introduced a variable flow rate of diluting air, by simply modifying its power voltage by using a rheostat.

Using a TAG based on dynamic mixing of two gas flows has the consistent advantage of greatly minimizing the errors associated with unwanted adsorptions of the target analyte onto the inner walls of all surfaces “washed” by the standard atmosphere. These adsorptions actually still occur, but in a pretty short time the equilibrium between adsorptions and desorptions establishes, and hence the concentration of the analyte within the standard atmosphere will not be affected anymore. However, the tubing connecting the various components of the TAG (made of PTFE Teflon, 0.25” i.d.) was kept as short as possible.

The concentration of HCN vapors in the final standard atmosphere, after mixing the two gas flows, is easily calculated using the relationship C_HCN_ = (C_0_·Q_1_)/(Q_1_ + Q_2_), where C_0_ = 10 ppm_v_ is the concentration of HCN vapors in the gas cylinder; Q_1_ is the flowrate of the gas flow delivered by gas cylinder, and Q_2_ is the flowrate of diluting air.

Seven different standard (test) atmospheres containing four ultra-trace (sub ppm_v_) and three trace (ppm_v_) concentrations of HCN vapors were prepared and further investigated by using the LCD-3.2E ToF IMS instrument: 100 ppb_v_, 200 ppb_v_, 300 ppb_v_, 600 ppb_v_, 1000 ppb_v_, 2000 ppb_v_, and 10,000 ppb_v_, respectively. Table 1 summarizes the experimental conditions used for generating the abovementioned standard atmospheres. A complete mixing of the two gas flows, which ensures a homogenous composition of the standard atmosphere, was sought by using both the mixing and the buffer chambers. 

Methyl salicylate (MSAL, C_8_H_8_O_3_, CAS No. 119-36-8) with a 99.9% purity, used as chemical standard, was purchased from Acros Organics BVBA (Fisher Scientific, Loughborough, UK).

## 3. Results

Experimental data generated by the hand-held ToF IMS instrument model LCD-3.2E as ion mobility spectra were recorded sequentially for each of the seven concentration levels of hydrogen cyanide. The experiments were run in triplicate during different days, and standard deviations between 6% and 2% were noticed for each HCN concentration.

The results of our investigation are summarized in Table 2, where C_HCN_ is the concentration of HCN in the standard atmosphere (calculated assuming an initial concentration of 10 ppm_v_ HCN inside the pressurized gas cylinder; see Table 1).

Ion mobility spectrometric response consisted of simple spectra, where the single product ion peak (PIP) that was noticed in the negative ion mode, at a drift time t_d_ = 4.26 ms, can be assigned to hydrogen cyanide HCN vapors. The negative reactant ion peak (NEG RIP) was observed at a drift time t_d_ = 4.74 ms. 

**Figure 3:**
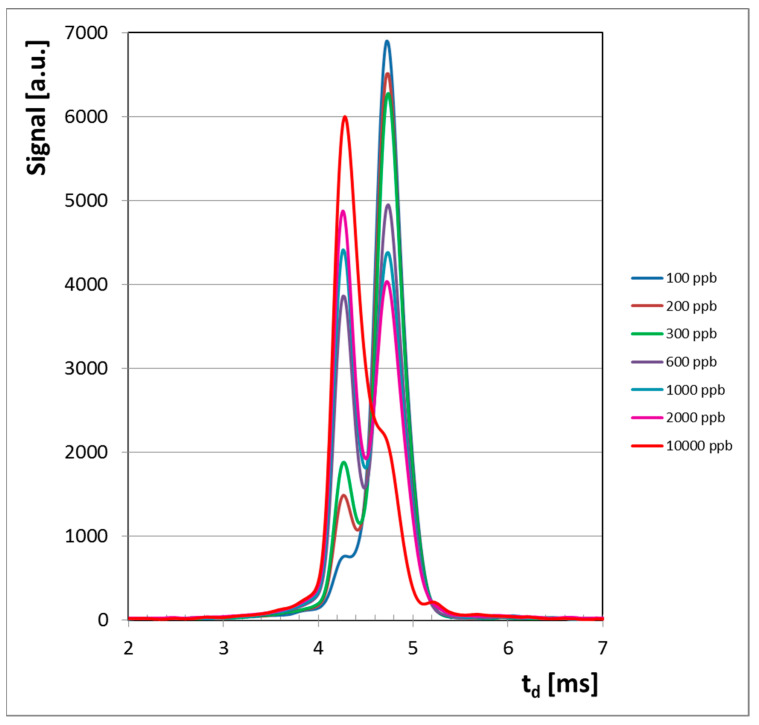
Ion mobility spectrometric spectra from HCN, obtained in the negative mode. Note: although all ion mobility spectra are collected from 1 to 20 ms, in order to increase the clarity only the useful part of the spectra (the temporal interval that includes all peaks, from 2.00 to 7.00 ms) is shown.

**Figure 4:**
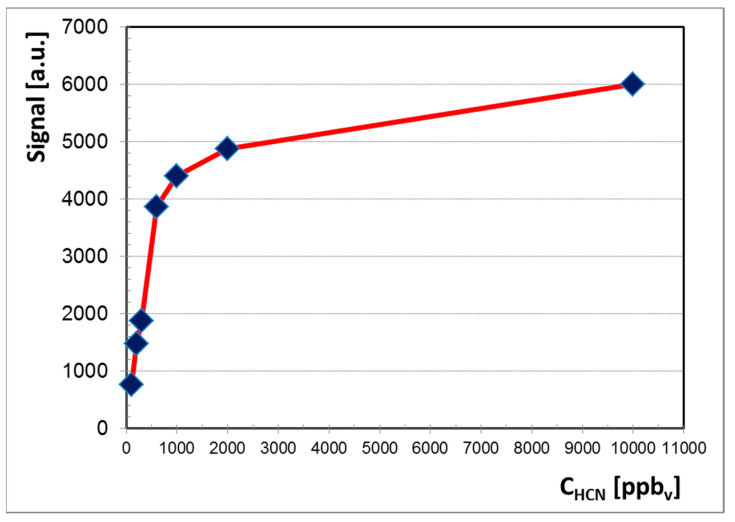
Calibration graph for HCN in the negative ion mode, normalized for the background air; peak height of the single product ion peak PIP is plotted.

All ion mobility spectra obtained in the negative mode are presented in Figure 3 and they clearly illustrate the conservation of electrical charge with the increase of the HCN concentration. In other words, when concentration of HCN increases, the intensity of the negative RIP (reactant ion peak) decreases, while the height of product ion peak (PIP, generated by HCN analyte) increases. Quantitative data were plotted as well, in order to obtain the calibration curve and finally to assess the quantitative response of the LCD-3.2E hand-held ion mobility spectrometer to vapors of HCN. The resulting calibration curve is presented in Figure 4.

Ion mobility spectra enclose valuable qualitative information, which is contained within the drift time t_d_ of an ion and the afferent reduced ion mobility K_0_. Table 3 summarizes this qualitative information; negative reactant ion peak (RIP) and product ion peak of HCN are displayed. One may easily observe that the HCN product ion has higher reduced ion mobility, compared to the negative RIP, which means that HCN forms a product ion that is smaller and/or more compact than the reactant ion cluster; a similar behavior was noticed previously for chlorine [6] and for carbon disulfide [8].

Reduced ion mobility was calculated by using the so-called “IMS cell constant”; this methodology has, among others, the advantage that it takes into account even the subtlest “fringe effects” produced by slight inhomogeneities in the DC electric field that propels the ions through the IMS cell. In addition, the use of the cell constant excludes the necessity of knowing or measuring both the instrumental parameters (such as drift length l_d_ and the electric field intensity E) and environmental parameters (temperature and pressure inside the measurement cell) [6,7]. Methyl salicylate (MSAL) was utilized extensively as a chemical standard in the negative operation mode, having a known reduced ion mobility K_0 of standard (MSAL)_ = 1.474 cm^2^ V^−1^ s^−1^. Ion mobility cell constant (noted with A) represents the product between K_0_ and drift time of the MSAL product ion. In other words, in order to calculate the reduced mobility of the HCN peak, the simple relationship A = K_0 of standard (MSAL)_ · t_d of standard (MSAL)_ = K_0 of analyte (HCN)_ · t_d of analyte (HCN)_ is used. Since t_d of standard (MSAL)_ = 6.88 ms, then the cell constant will be: A = 1.474 cm^2^ V^−1^ s^−1^ × 6.88 × 10^−3^ s = 10.141 × 10^−3^ cm^2^ V^−1^. 

The ratio between the drift times of PIP and RIP, which is equivalent to the ratio of reduced mobilities, has also its relevance: t_d PIP_/t_d RIP_ = K_0 RIP_/K_0 PIP_ = 0.898. In other words, this is a normalization of product ion drift time against the drift time of negative reactant ion peak.

Resolution of the LCD-3.2E IMS instrument, defined as the ratio between the drift time of a certain ion and its width at half height (R_IMS_ = t_d_/Δt_d_), was calculated for negative RIP ion and for PIP ion generated by HCN; the results are presented in Table 4.

This value for resolution—around 15—is typical for commercial hand-held IMS instruments equipped with a miniaturized measure cell, which is definitely the case of the LCD-3.2E instrument. A better resolution would be beneficial for sensitivity as well; most probably, lower concentrations than our lowest measured level of HCN (100 ppb_v_) could be determined if peaks’ width decreases; in other words, peak overlapping is a significant problem, especially at very low HCN concentrations (<0.1 ppm_v_).

## 4. Discussion

The logarithmic aspect of the calibration curve observed in Figure 4 is very characteristic for any IMS response obtained using a radioactive ionization source [1]. Therefore, we may infer the similarity of the quantitative IMS response generated by the hand-held ToF IMS system with nonradioactive ionization source that was used by us (the LCD-3.2E) to the quantitative response produced by any ToF IMS device equipped with a radioactive source [4,5,6,7,8].

The IMS spectra for higher concentrations of HCN (>2 ppm_v_) in the negative ion mode show that the saturation threshold was not reached. Moreover, ion mobility spectra at the highest HCN level investigated, of 10 ppm_v_ (11.2 mg m^−3^), indicate that saturation was not reached, since the reactant ion peak is still present at about 25% of its initial value. When saturation is reached, the whole amount of negative reactant ions has been consumed and, as a consequence, the reactant ion peak RIP disappears from the IMS spectrum. We must emphasize that saturation of any ion mobility spectrometer must be avoided, because it usually leads to a heavy contamination of both the IMS measurement cell and other inner surfaces that come in contact with the analyte; this contamination will finally produce unwanted memory effects.

The possible interferences are always a concern, and this is, of course, valid for the IMS technique as well. Since, however, in IMS only a relatively small percentage of chemicals (approximately 20%) generate negative ions, this means that a certain degree of selectivity is achieved by negative ion formation itself. In other words, a negative ion spectrum will definitely be cleaner (with a smaller number of peaks) and less prone to interferences than a positive ion spectrum.

A careful examination of the quantitative response (Figure 4) and of ion mobility spectra obtained for all the investigated concentration levels (Figure 3) allows us to conclude that:

Minimum measured concentration was 100 ppb_v_ (0.11 mg m^−3^) of HCN vapors.

Linear dynamic range is from 100 ppb_v_ (0.11 mg m^−3^) to 600 ppb_v_ (0.67 mg m^−3^) HCN.

Saturation is thought to occur at approximately 20 ppm_v_ (22 mg m^−3^) HCN, which is in good accordance with the fact that the dynamic range of an ion mobility spectrometer extends to about two orders of magnitude.

During our investigation, saturation threshold of the IMS instrument, indicated by the total disappearance of the negative reactant ion peak, was not reached. Therefore, lack of contamination of the IMS cell was fortunately achieved, and, thus, we may fully rely on the quantitative data provided by the IMS instrument.

All ion mobility spectrometric responses for HCN were obtained in the negative ion mode. This way, an ionization-based selectivity is achieved, since much fewer substances produce negative IMS responses compared to those that generate positive IMS spectra. Vapors of hydrogen cyanide did not produce any response in the positive ion mode. The LCD-3.2E instrument responded in real time (several seconds) to vapors of hydrogen cyanide.

Assigning an identity for ions that are generated in the cell of any IMS instrument is a difficult task, which could be efficiently solved only by using a hyphenated IMS-MS analytical chain. Such complex hyphenated IMS-MS devices were already used, especially with the purpose of investigating the identity of ions generated by chemical warfare agents, illegal drugs, explosives, or highly toxic chemicals such as chlorine and phosgene [6,7]. Assigning the ions produced by HCN vapors inside the IMS cell with nonradioactive ionization was, therefore, not feasible in this study. Nevertheless, we can make the reasonable assumption that, in the negative ion mode, the product ions that are generated by hydrogen cyanide may be considered as cyanide ions, most probably clustered with neutral species such as water, carbon dioxide, or nitrogen. Recent studies have reported the formation of CN^−^ and OCN^−^ ions from vapors of hydrogen cyanide [18].

Reduced ion mobility for the single ion peak generated in the negative mode was found to be K_0_ = 2.38 cm^2^ V^−1^ s^−1^, a value that is in good accordance with other results indicated in the literature: 2.38 cm^2^ V^−1^ s^−1^ [19], 2.46 cm^2^ V^−1^ s^−1^ [20], or 2.47 cm^2^ V^−1^ s^−1^ [18]. Figures of merit given in the literature and those found by us are summarized in Table 5, where LoD is the limit of detection and LoQ the limit of quantification, respectively. 

Unlike the aspiration-type IMS systems, where the detection sensitivity towards HCN is poor (a response is elicited only at very high levels of HCN vapors, of several hundreds of ppm_v_) [21,22,23], the classical (ToF) IMS devices equipped with either nonradioactive [18,24,25,26] or radioactive [19,20,27] ionization sources showed similar analytical performances (as LoD, LoQ, measurement range). The only notable exception is the pretty poor LoD of 10 mg m^−3^ reported in [26], which could be explained, very probably, by errors that occurred when generating the standard atmospheres with HCN vapors using chemical reaction between a solution of cyanide and an acid.

The analytical figures of merit related to HCN detection and quantification are very similar, for instance, to those reported by us recently for another toxic industrial compound—carbon disulfide—using a ToF IMS with tritium radioactive ionization source [8].

**Table 5:** Quantification of HCN using various IMS systems. RS—instrument equipped with a radioactive ionization source; NRS—instrument equipped with a nonradioactive ionization source.

Instrument	K_0_ (cm^2^ V^−1^ s^−1^)	LoD	LoQ	Ranges	Ref.
ToF-MS with PI (NRS)	-	-	0.5 ppb_v_	-	[24]
DT-IMS with AEE source (NRS)	2.47	0.057 mg m^−3^	-	Linear range: from 0 to 10 mg m^−3^. Calibration: from 0 to 50 mg m^−3^	[18]
DT-IMS & FAIMS (new FAT IMS) (RS)	2.38	0.16 ppb_v_	-	-	[19]
ToF IMS, model SABRE-4000 (RS)	2.46	-	0.050 mg m^−3^	From 0.02 to 10 mg m^−3^	[20]
ToF IMS, model GDA-2 (RS)	2.52	-	-	1 ppm_v_; 10 ppm_v_; 100 ppm_v_	[27]
ToF IMS, model LCD-3.3 (NRS)	2.26 and 2.33	-	0.15 mg m^−3^	Calibration: from 0 to 60 mg m^−3^ Saturation: *ca*. 15…20 mg m^−3^	[25]
ToF IMS, model LCD-3.2E (NRS)	-	≈10 mg m^−3^	-	Range measured: 10 to 100 mg m^−3^	[26]
aIMS, model ChemPro 100 (RS)	-	-	-	Responded only at high levels of HCN vapors (>1000 mg m^−3^)	[21]
aIMS, model M90 (RS)	-	>415 mg m^−3^	-	-	[22,23]
Current study: ToF IMS, model LCD-3.2E (NRS)	2.38	20 ppb_v_	67 ppb_v_	Calibration: from 0.1 to 10 ppm_v_ Linear range: from 0.1 to 0.6 ppm_v_ Saturation: *ca*. 20 ppm_v_	-

### Validation

The validation process was simple and was accomplished in order to quickly assess the adequacy of the IMS-based analytical method to the quantification of HCN vapors. The following main parameters were evaluated: sensitivity, S; limit of detection, LoD; limit of quantitation, LoQ; linear range of response; accuracy; trueness.

Standard deviation (s_d_) of the background noise, which gives the background signal, was calculated by utilizing the 500 datasets for the drift times from 10.00 to 20.00 ms (in increments of 0.02 ms) for all the eight ion mobility spectra; finally, the average was s_d_ = 10.3 a.u. Furthermore, LoD was calculated as the concentration of HCN that produces a response that is equal to 3·s_d_, while LoQ is the concentration of HCN that generates a response equal to 10·s_d_. Sensitivity was calculated as the ratio between the response variation and concentration change S = ∆Y/∆C. We have to indicate that only the linear range response (from 100 to 600 ppb_v_ HCN) was considered. Analytical figures of merit for HCN quantification are provided in Table 6.

Precision was assessed by realizing the analyses in triplicate, as indicated in Table 2, while accuracy was ascertained by relative standard deviation (RSD). For the product ion generated by HCN, the calculated RSD was found to be from 2% to 6%; therefore, having a RSD < 10% we may claim a good repeatability of our results.

Traceability to a standard, certified reference material (CRM) (the standard atmosphere with 10 ppm_v_ ±10% of HCN in N_2_, in gas cylinder) was accomplished. This way, by using this CRM, the trueness of the obtained results is supported.

## 5. Conclusions

Fast detection and quantification of the toxic compound HCN has been proven, at trace (sub-ppm_v_) levels, using a portable, lightweight, hand-held ToF IMS instrument with nonradioactive (corona discharge) ionization source, model LCD-3.2E (Smiths Detection Ltd.). Hydrogen cyanide vapors were detected and quantified in real time (seconds), in the negative ion mode. Simple spectra that included only one product ion peak assignable to HCN, with a reduced ion mobility K_0_ = 2.38 cm^2^ V^−1^ s^−1^, were obtained. This product ion peak generated by hydrogen cyanide is located very close to the negative reactant ion peak (with K_0_ = 2.14 cm^2^ V^−1^ s^−1^). It is worth mentioning that HCN is one of the very few chemicals that generate product ion peaks having a shorter drift time (and hence a higher ion mobility) than the reactant ion peak in the negative operating mode; other such compounds are chlorine, phosgene, and carbon disulfide.

A seven-point calibration curve for HCN vapors, from 0.1 to 10 ppm_v_ mg m^−3^, was built. A linear range from 0.1 to 0.6 ppm_v_ was observed. Detection limit was found to be 20 ppb_v_ HCN. Our findings were in good agreement with the results reported previously in the literature. Minimum measured concentration of HCN was 0.1 ppm_v_, which is 100 times lower than the OSHA PEL-TWA limit for 8 h (10 ppm_v_), *ca*. 50 times lower than the TLV and REL-STEL (4.7 ppm_v_), and 500 times lower than the IDLH (50 ppm_v_). In other words, fast detection and quantification of HCN vapors could be successfully used in industrial hygiene, in order to protect health and life of the workers. Saturation of the response is thought to appear at concentration levels above 20 ppm_v_ HCN.

Future research directions that could be approached are either investigations on HCN vapors using different types of ion mobility spectrometers, or the attempt to increase the sensitivity and resolution of the IMS response by using dopants, in order to alter the ionization chemistry in the negative ion mode, and, subsequently, to shift the negative reactant ion peak towards longer drift times.

We conclude that the robust, hand-held, lightweight ToF IMS instrument equipped with a nonradioactive (corona discharge) ionization source, model LCD-3.2E manufactured by the company Smiths Detection Ltd., displays a very good sensitivity and a fast response for HCN vapors present in air at trace (sub-ppm_v_) levels. Therefore, it has been proven again that ion mobility spectrometry is definitely an extremely valuable tool in protecting the health and life of either workers (industrial hygiene), soldiers (on the battlefield), or civilians (in chemical terrorist attacks). 

## Figures and Tables

**Table 1:** Concentrations of HCN vapors in the final standard atmospheres, correlated to the two gas flow rates.

C_HCN_ (ppb_v_)	Gas Flowrate with C_0_ = 10 ppm_v_ HCN Q_1_ (cm^3^ min^−1^)	Diluting Air Flowrate Q_2_ (cm^3^ min^−1^)
100	40	4000
200	80	4000
300	120	4000
600	130	2000
1000	110	1000
2000	130	500
10,000	200	0

**Table 2:** Summary of quantitative results obtained using the LCD-3.2E hand-held ToF IMS instrument in negative ion mode (three replicates were used for peak height, in order to calculate standard deviation). Legend: NEG RIP—negative reactant ion peak; PIP—product ion peak from HCN.

C_HCN_ (ppb_v_)	IMS Data—Negative Ion Mode	
	Drift Time t_d_ (ms)	Peak Height h_max_ (a.u.)
0	NEG RIP 4.74	8500 ± 160
100	PIP 4.26	760 ± 40
200	PIP 4.26	1480 ± 55
300	PIP 4.26	1880 ± 62
600	PIP 4.26	3860 ± 78
1000	PIP 4.26	4400 ± 90
2000	PIP 4.26	4880 ± 120
10,000	PIP 4.28	6000 ± 135

**Table 3:** Reduced ionic mobilities K_0_ calculated for ions produced by HCN.

Operation Mode	Ion Drift Time, t_d_ (ms)	Reduced Ion Mobility ^1^, K (cm^2^ V^−1^ s^−1^)	Reduced Ion Mobility ^2^, K_0_ (cm^2^ V^−1^ s^−1^)
NEGATIVE	RIP: 4.74	2.144	2.139
	PIP: 4.26	2.386	2.381

^1^—Calculated by the IMS software TrimScan. ^2^—Calculated using the IMS cell constant (A): K_0_ = (A/t_d_).

**Table 4:** Resolution of the LCD-3.2E IMS instrument for HCN.

Ion Drift Time, t_d_ (ms)	Peak Width Al Half Maximum, Δt_d_ (ms)	Resolution, R_IMS_
Neg RIP: 4.74	0.32	14.8
PIP for HCN: 4.26	0.28	15.2

**Table 6:** Analytical figures of merit related to IMS detection of HCN in the negative ion mode.

Ion Mode	LoD (ppb_v_)	LoQ (ppb_v_)	Linear range (ppb_v_)	Equation	R^2^	S (a.u./ppb_v_)
Negative	20	67	67—600	Y = 6.1286·X + 156.4	0.9955	6.2

## Data Availability

Data available to the authors.
